# Exploring the Opportunity to Use Virtual Reality for the Education of Children with Disabilities

**DOI:** 10.3390/children10030436

**Published:** 2023-02-23

**Authors:** Ioana Bianca Chițu, Alina Simona Tecău, Cristinel Petrișor Constantin, Bianca Tescașiu, Tamara-Oana Brătucu, Gabriel Brătucu, Ioana-Mădălina Purcaru

**Affiliations:** 1 Faculty of Economic Sciences and Business Administration, Transilvania University of Braşov, Universității Street, No. 1, Building A, 500164 Braşov, Romania; 2 Faculty of Psychology and Educational Sciences, Transilvania University of Brașov, N. Bălcescu Street, No. 56, 500164 Braşov, Romania; 3 School Center for Inclusive Education Brasov, 13 Decembrie Street, No. 125, 500164 Braşov, Romania

**Keywords:** sustainable inclusive education, virtual reality (VR), modern teaching technologies, children with disabilities, interactive learning environments, teaching and learning strategies, education digitization

## Abstract

Inclusive education represents one of the most challenging aspects of modern education. In recent years, a major challenge in achieving inclusivity in education has been to find modern tools that might adapt the teaching process to the needs of children with disabilities. This article investigates the possibility to use virtual reality (VR) technology to improve the learning process of children with disabilities. In this regard, a qualitative study based on the Focus Group method was conducted among 31 specialists who work with children with disabilities, with the aim of identifying potential opportunities and limits of using VR technology in the education of this category of children. The research results reveal that for most of the interviewees the VR application was a new experience; they appreciated that this technology could improve the educational process for children with disabilities and it could become a useful tool to support the education of such children. These results could have a positive impact on the activities carried out by policymakers, academia, and the economic environment in their efforts to implement policies aimed at improving the inclusive education system. To this end, some VR applications could be developed in a collaborative system involving IT companies and universities in the designing and implementation of these applications.

## 1. Introduction

Inclusive education provides education for all categories of children (including children with disabilities), and it is considered one of the most effective ways to combat discrimination and ensure equal opportunities for all children. In Romania, the new legislative framework of education is based on two main principles, as follows: 1. the principle of non-discrimination—according to which access to quality education is achieved without any discrimination; 2. the principle of ensuring equal opportunities—according to which the differences regarding access and treatment for all the beneficiaries of education without any discrimination are removed [1]. At the beginning, the concept of inclusive education referred to children with disabilities in the regular school system, but the concept has evolved and now it is defined by UNESCO as “the process of strengthening the capacity of the education system reach out to all learners” [2]. Thus, providing equal opportunities represents one of the greatest challenges of inclusive education [3,4]. To work in a common environment, to reach all students and to include all the children, inclusive education uses specific tools and technology to help both teachers and children in reaching the expected results. Tools such as “secularity, unity, and equality” [5] can significantly contribute to achieving a sustainable inclusive education; the key to accomplishing this goal is represented by the teachers and their positive attitudes regarding this aspect [6].

Among the new technologies, the immersive technologies—such as Virtual Reality (VR) or Augmented Reality (AR)—are more and more used, both in the field of computer technology [7] and also in other fields [8,9,10,11,12]. Vaughan, Gabrys and Dubey [13] identified five key fields in which VR training and assessment had a significant increase: the medical field, industrial and commercial training, rehabilitation, and remote training.

A series of studies investigated the influence of technologies on learning and education. According to Hsin, Li and Tsai [14], about one-third of the studies published on the Web of Science between 2008 and 2013 that considered young children’s learning approached the following topics: children from immigrant families, children with low socioeconomic status or children with special needs. Most of them highlighted the positive effects of technologies, because technologies lead to the development of multiculturalism, to the increase of interactions between children and to their collaboration [15,16]. Other studies revealed that children with disabilities, especially from Northern countries, are thought to be the main beneficiaries of this “digital revolution” [17]. Specialists claim that these children can benefit from appropriate learning materials and overcome communication barriers with the help of “cyber learning techniques and environments” [18,19,20]. According to UNICEF, at least 93 million children with disabilities “are one of the most marginalized and excluded groups in society and they suffer from discrimination with regard to access to education” [21]. It is not easy to provide the right education for children with disabilities, as they face specific barriers, and have access to limited social and economic opportunities [22]. Furthermore, these children are not just consumers of technology, but also creators, a role that is still not sufficiently explored [14].

In this context, the aim of our research was to identify opportunities and limits of using VR as a powerful tool in achieving sustainability in inclusive education. To this end, exploratory research was conducted to find out from specialists working in the field what the main aspects are that could make the use of VR a possible solution to be used in schools to improve inclusive education. The specialists were selected from schools in the Transylvania region of Romania and were interviewed by using the Focus Group method.

The main questions of the research were: (1) How familiar are the specialists who work with children with disabilities with VR technology in education? (2) Is it appropriate to use technologies such as VR for education of children with disabilities? (3) Who are the most suitable children with disabilities to use VR in their education?

Obtaining the answers to the above questions could help to identify possible solutions to achieve the best educational tools that could be used to help children with disabilities in the learning process. The article is divided into five parts: introduction, theoretical background, materials and methods, results and discussion and conclusions and implications.

## 2. Theoretical Background

### 2.1. Inclusive Education and VR Technology

According to several authors, the aim of inclusive education is to improve diversity and to ensure equal access to education and to all educational programs and environments for all students [23,24,25]. They stated that all students, with or without disabilities, should be in the same classroom [2,3,21,22]. Ainscow, Slee and Best [26] support the idea that “every learner matters equally” and he/she has the right to receive effective learning opportunities. They argue for the need to focus regular school on inclusive education, advocating a “whole system approach” organizational model. This system only works if the process of inclusion takes place in several environments, namely in the community, school, and classroom, with the support of teachers and students [27]. Ainscow also argues that to become effective, inclusive education needs to support all the pupils [28]. With this aim, he proposes a concept called “inclusive inquiry”, which refers to the involvement of both teachers and students in a continuous dialogue about the main ways to improve the educational processes for better inclusion [29].

Inclusive education is based mainly on the principle according to which the educational system must include all the children and the schools must be prepared to respond to the individual needs of these children [30,31,32].

According to Cretu and Morandau [33], it is very important to prepare teachers for addressing a diversity of pupils and for developing inclusive educational environments for all in order to assure a sustainable future and equal opportunities for all the students. Globally, significant steps have been taken to empower teachers and future teachers to work in inclusive education. Some authors note that inclusive education is a never-ending process that always requires improvements and new tools. These tools should not only improve the situation at some point, but they would also bring major changes in education [34].

Since the role of inclusive education is to create a proper learning environment, several authors have proposed the use of organized role-playing games [35], where the learning style could evolve from passive learning to interactive learning by involving the “learners” as active participants. Therefore, they can achieve cognitive skills [36]. A series of studies revealed that interacting with an avatar, in an environment simulated through virtual reality, is more realistic and useful [37], thus contributing to the creation of a more accessible learning environment. Starting from Gibson’s Affordance Theory of accessibility, several specialists concluded that VR could make the learning environment more accessible [38,39]. Learning by exploring, obtaining information in real time, using e-platforms, self-discovery, experimenting, and testing are just a few of the new elements that can be used in education. Furthermore, the use of technology could enhance these educational tools. For example, computers can be used in activities such as teaching, exercises, exploring, multimedia projects, or career guidance [40,41]. Several studies reveal that using virtual environments to transform social interrelations might improve learning [42] and they also might contribute, to some extent, to the concept of “equal opportunities” in education [43].

VR may become helpful in the education of children with disabilities, by enabling pupils and students to form and improve social and emotional abilities [44,45,46,47]. In this sense, it is useful to present some initiatives, such as a virtual reality program created to improve the emotional and social adaptation skills of children with Autism Spectrum Disorders, who were exposed to six learning scenarios that were presented through a four-side immersive virtual reality environment also known as “Half-Cave” and their movement was tracked [45]; another proposed application was a computerized visualization system, which automatically determines the child’s emotional state [47].

In recent years, VR technology has become more and more present in our society, in fields like e-commerce, education, services, and medicine and is becoming more accessible to everyone [48]. The concept of “virtual reality” can be described by using the following related terms: “reality”—the actual world, which involves the physical presence of people and objects; “virtual reality”—a digital representation of the world; “virtually”—a virtual presentation of a possible world, based on the rules of the real world [49]. There are several definitions of VR—for instance, VR might be defined as “a system in which users feel that they are in a virtual world with various equipment and the users interact with this world” [50] or “VR leverages immersive technologies simulates interactive virtual environments or virtual worlds with which users become subjectively involved and in which they feel physically present” [51]. VR is a communication environment that involves the existence of four elements: a virtual world, immersion, sensory feedback, and interactivity [52]. The objective of VR is to offer an authentic experience to users by placing them in a safe and controllable environment. The increasing affordability of IT applications is creating favorable conditions and offering increasing opportunities to use VR in educational processes [53], thus being able to respond to the different learning needs of people with disabilities [20]. Two attributes of VR can contribute to increasing the accessibility of the education process—“the sense of presence, and the embodied affordances of gesture and manipulation in the 3rd dimension” [54].

Using technological tools to make learning more dynamic, more efficient, and more participatory might improve inclusive education [55]. In inclusive education systems, VR might transform children’s learning style by providing more challenges, it can attract and focus children’s attention, it can offer proper control of the learning environment, and, also, it can involve the participants emotionally [56]. To this end, virtual reality, and machine learning were used to create a learning platform capable of solving the socialization problems by exposing the children to social lessons, such as “a route to school”, “behavior in computer class”, “interaction with peers”, or “safety skills” [56].

This may also be valid for children with disabilities, especially for those with intellectual disabilities who refuse real experiments and interactions [57], because VR could provide immersion and user interaction with a virtual environment [58]. In this way, a description of the real world without any imaginary components may be offered, abstract concepts can be presented through visual aids, and children may be positioned in certain contexts in which they cannot be positioned in real conditions (because of different social constraints, resources or constraints directly related to disability) [56]. For example, for children with autism, VR provides authenticity and realism in a controlled environment, so it can enhance learning and perception processes, enabling the acquisition of real-world skills [59]. Video modelling (“video-based instruction”) through VR might become helpful for autistic children, children with different intellectual disabilities, or children with developmental disabilities, by increasing their degree of involvement [57,60]. At the same time, using VR technology offers the possibility to repeat presentations and to do different exercises without involving a human interaction [60]. Different studies reveal that VR technology might help people with neurodevelopmental disorders to develop communication abilities, as these persons can “navigate” in an easier way in a virtual environment that provides challenges similar to the real world [61].

Using virtual environments created with VR technology can facilitate learning about logical-mathematical concepts or even leisure activities, such as avoiding unintentional injuries [62]. VR can be used to “increase self-esteem and sense of empowerment” [63] and prevent depression by helping to overcome disability barriers [64]. Finally, the interaction and communication between children and teachers could be improved [65,66].

Nevertheless, scholars identified a series of disadvantages that may prevent the use of VR in education: non-realistic representation, too little flexibility, costs, user discomfort, etc. [67]. Sometimes, problems, such as speed reading must be taken into consideration when VR is used [68]. Thus, the role of specialists in educating children with disabilities becomes very important. They must constantly find new ways to improve children’s learning performance, help them become more independent and improve their social skills, and new technologies (such as VR) can help them achieve this goal [69].

### 2.2. Inclusive Education in Romania

In Romania, the educational system is based on values such as equity, respect for the right of each student to have equal chances of access, participation and reaching the optimal potential, as well as the principle of inclusion, which implies a permanent improvement of the services offered to all the members of the community, regardless of individual characteristics, by participating, developing adaptive behaviors, developing cognitive skills, building positive affective relationships, and ensuring well-being, with an emphasis on individual needs. Special education and inclusive education are important components targeted in the process of adapting Romanian education to European trends.

The implementation of inclusive education in Romania started in 1989, after the fall of communism, which transformed the country into a modern one based on care for the community and its children. Promoting and guaranteeing all children’s rights, as well as finding new ways to ensure equal opportunities have become priorities in Romania, by respecting diversity and ensuring access to education and social life for all categories of children. Special and inclusive education is an integral part of the Romanian national education system with the aim of offering all children/students/young people educational programs adapted to the degree of disability and their development needs. It is desired to be a viable, open, flexible subsystem, capable of adapting to Romanian social requirements [70]. The school organization and the curricula are flexible and adaptable to each child’s situation, allowing each child to progress at their own pace and to be treated according to their learning abilities. For achieving this goal, it is necessary that the objectives, the establishment of the training contents, the ways of transmitting the information in the classroom, and the evaluation of the students be done differently.

The Ministry of Education supports the transition of all schools to a differentiated pedagogical system that guides the customization of the pedagogical strategy for each pupil/student, according to his needs, rather than identifying only students with special educational needs (SEN). Thus, according to the draft of the new education law, the education system will offer these students support on five levels, as follows: the first four levels aim at an educational intervention in mainstream schools, and the fifth in special schools.

Inclusive support level 1 is a form of inclusive education where the teacher can provide educational services alone, in ordinary teaching conditions, in the form of individual support activities. Level 2 inclusive support requires the regular intervention of specialists to support the teaching–learning process; this process can be carried out entirely in the regular classroom but requires the presence of a support teacher. Level 3 inclusive support involves certain support activities that cannot be carried out in the ordinary classroom under normal conditions and that need individualized interventions by specialists to reach the maximum potential for the pupils’/students’ development. It consists in working with individualized educational support or with small working groups under the surveillance of support teaching staff, during and outside of class hours. Level 4 inclusive support involves an increased level of support and complex and integrated individualized interventions.

The pupils/students are the beneficiaries of a differentiated curriculum and special programs; they carry out between 80% and 90% of learning activities in dedicated spaces within educational units, they can work in small groups, and they participate in limited general education activities, depending on their profiles. Level 5 special support is a form of special education, and the main responsibility for its implementation rests with the teaching staff in special education units. It is provided to those students whose growth, development, or learning objectives cannot be achieved through inclusive support measures [1,71].

## 3. Materials and Methods

Considering the research questions, and based on the literature review, the research objectives were established as follows: (O1) Identifying the level of familiarization with the VR technology among specialists that work with children with disabilities. (O2) Exploring the opportunities to use VR technology in the education of children with disabilities. (O3) Identifying specialists’ views on children with disabilities who are best suited to use VR in learning. In order to achieve these objectives, a qualitative study that included four focus groups was conducted. It included specialists (teachers and support teachers) who are working with children with disabilities in the education system.

### 3.1. The Sample Structure

The sample members were selected from the four most important inclusive education school centers in Transylvania. To identify them, the official list of teachers and specialists employed in these school centers was consulted. The school managers were contacted to propose the participants for the group interviews. They were contacted by phone in order to obtain their consent to participate in the research. Those who agreed were kindly asked to answer a few pre-selection questions. In total, 32 respondents who fulfilled the selection criteria expressed their agreement to participate in the study. At the end, one of the selected members was not able to participate in the interview.

A total sample of 31 participants was established based on the following selection criteria: (1) type of specialization: speech therapists, psychologists, special education teachers, itinerant/support teachers; (2) years of professional experience in working with children with special needs (minimum of 5 years of experience); (3) the types of disabilities that teachers have experience working with (teachers who work with children who cover a wide range of disabilities were selected)—mental disorder (moderate/severe), some forms of autism (that can be considered disabilities): Down syndrome, hyperkinetic syndrome, cerebral ataxia, Stargardt disease, schizoid elements, epilepsy, physical disabilities, severe hearing loss, physical disabilities, sensory–auditory deficiencies, motor disabilities, spastic tetraparesis, Dandy–Walker syndrome, severe associated deficiencies, physical/motor deficiencies, mental retardation, epilepsy, microcephaly.

The sample structure is presented in Table 1.

The 31 participants (of which 26 are women) have an average age of 43 years and an average of 15 years of experience. They were divided into four groups according to their specialization, as follows:

- Group 1: 8 psychologists with a particular focus on speech therapy;
- Group 2: 8 teachers involved in special education;
- Group 3: 8 specialists in physical therapy, teachers of physical education and sport, special education teachers;
- Group 4: 7 itinerant/support teachers.

The sample might be considered relevant, as the total number of such teachers is generally low in the considered region.

### 3.2. Method and Data Collection

The focus groups conducted in this qualitative study were based on a semi-structured interview guide, and they took place during January–February 2020, with sessions of 90–120 min, organized in a special room at the School Center for Inclusive Education Brasov; the room was arranged and equipped with specific equipment for group interviews, and it benefited from technology for testing VR equipment.

All the four interview sessions were moderated by two of the authors of the article, researchers with expertise in qualitative research, marketing, and psychology. At the beginning, the rules of discussion were presented, so all the participants could feel comfortable and free to express their opinions. They were informed that the discussions would be audio-recorded so that they could later be transcribed as faithfully as possible. Participants were asked to consent to the use of their information for research purposes only. At the same time, the aspects regarding confidentiality were presented.

During the discussions, the moderators only got involved to guide the discussions and they avoided any personal judgments that might influence the participants’ point of view. In order to obtain as many spontaneous opinions as possible that accurately reflect the feelings or beliefs of the participants, they were encouraged to contribute to the discussions whenever they wanted without any restrictions; they were assured that all experiences and opinions were equally valuable for the research purpose.

After the participants were informed about the research purpose—to identify ways that advanced technologies such as VR could improve the educational process of children with special needs—information about the proposed technologies was given (what do they represent, how do they work, in what fields are they used etc.). For the VR technology, we used the Samsung Gear VR (a virtual reality headset). It comprises a stereoscopic head-mounted display (providing separate images for each eye), stereo sound and head motion tracking sensors (gyroscopes, accelerometers, etc.).

To become familiar with the VR technology, each participant experienced virtual reality with the Samsung Gear VR glasses using images or little movies offered by a free application. In order to understand the various possibilities of exposure through VR, the participants watched the following demos: a visit to the museum, a visit to an animal farm theme park, access to the seashore, and watching images from space.

After this preliminary session, the group interviews were conducted using a semi-structured interview guide, which contains open questions aimed to reach each research objective. The main questions of the interview were the following: How familiar were you with VR technology before this presentation? How do you appreciate this VR experience? How do you think VR could be used in the educational process of children with disabilities? Let’s imagine various situations where VR would help in the educational process. How should this technology be used to be efficient in the case of children with disabilities? Among the children you have worked with so far, can you identify a category that would appreciate VR/for which VR could be used in an educational activity?

The discussions were audio-recorded, transcribed, and analyzed using the content analysis [72]. The “context units” were used and “thematic distinctions” were considered according to Krippendorff [73]. Thus, the discussion transcript was analyzed to find the most relevant themes for the research objectives. Content not relevant was excluded (descriptions with no connections to the educational process). The content analysis process began with familiarization with the data by listening to the recordings and reading the transcripts [74]. The next step was to identify the main topics by analyzing each discourse in depth. Finally, the most relevant topics obtained by “thematic distinction” [73] were integrated in the research report. Utterances with similar meanings, even if expressed in different words or at different times, were included in the same category of answers. “Data triangulation” was used to verify the research validity [75]. In this respect, the results were compared with the four school managers’ opinions and the results of other similar studies about the use of VR presented in relevant literature [20,53,54,55,56,57,58,59,60,61,62,63,64,65,66,67,68,69].

## 4. Results and Discussion

The research results and discussions were grouped considering the research objectives. These results provided an in-depth understanding of the research problem, considering the benefits of exploratory research, which allows for a better understanding of the human experience [76].

### 4.1. (O1) Identifying the Level of Familiarization with the VR Technology among Specialists Who Work with Children with Disabilities

The first research objective aimed to explore the knowledge/familiarity of the interviewed specialists with VR technology before the presentation made during the interview (as mentioned in the Methodology section) and in identifying the opinions regarding the experiences created by this one. In this sense, the participants were asked to present their previous experiences in using VR technology and their opinion about these experiences. Feedback of persons without previous VR experiences was also requested. For most of the interviewed persons, using VR represented a new experience, as it was the first time they had used VR special glasses. Half of the specialists had experienced VR only at the cinema (3D movies), and only two of them declared that they were users of similar VR equipment.

- Participants’ feedback after testing the VR technology

All respondents appreciated the experience created by the VR technology, and they described it as: “very interesting” (#1, #2, #4, #5, #6, #7, #8); “new and fascinating” (#1); “it develops powerful emotions” (#3); “it was very interesting, I would try it again. I felt very relaxed and anchored in the virtual reality” (#6); “as an immersion of senses” (#18); “an interesting experience, of updating with the new trends in technology” (#24).

### 4.2. (O2) Exploring the Opportunities to Use VR Technology in the Education of Children with Disabilities

Another research objective was to obtain the specialists’ opinions regarding the opportunity of introducing VR in the educational process of children with disabilities.

- Advantages and disadvantages of introducing VR technology into the educational process

As awareness of VR was quite low among the sample members, the discussions focused on the participants’ experiences following the use of VR at the beginning of the interview. The main opinions concerning the opportunity of introducing VR technology into the educational process revealed that: “It is a very interesting experience, because sometimes we can’t use concrete materials in our activity” (9); “an efficient way of transmitting information” (#10); “very useful in our work with children with disabilities” (#14); “a useful experience, that helps learning new things” (#23); ”useful, beautiful and necessary for all children” (#26); ”useful in a balanced development of children with disabilities personality” (#27); ”it offers the possibility to get information in a very pleasant way” (#13).

Some of the participants, besides highlighting positive aspects of VR, had some doubts about using this technology over a long time period, thinking that “it is tiring for the eyes” (#4); “technically speaking—it could be better” (#20); “it is an interesting experience, but it gave me the impression of an untrue reality” (#22). In this context, it would be necessary to improve the VR equipment and the quality of the movies watched in VR.

According to the specialists, a significant part of the methods and learning tools used with the typical children are not efficient in the education of children with disabilities. So, using new methods—i.e., using VR in the education of children with disabilities—could be considered a solution that facilitates the learning process of these children. According to the interviewed specialists, such children live in a quite different reality than the typical children and any attempt to make the educational process easier and more efficient is appreciated.

The participants highlighted the following advantages offered by VR: the ability to present information in an attractive way; the ability to virtually transport the children to some locations inaccessible for them; the ability to present realities otherwise unimaginable for these children.

Some of the specialists considered this technology useful for relaxation as it is capable of capturing children’s attention. For some of the children, the images could be accompanied by appropriate music to help them relax; for other children, who are in certain crises, immersion into a familiar environment could be useful. In this regard, one of the participants gave the example of a boy who loves horses and becomes calm in their presence. Since in the educational process he cannot be near horses, the virtual transportation close to his favorite type of animal would help him relax when he is nervous, or it could motivate him in other contexts.

One of the teachers of physical therapy appreciated that using VR technology could help children to exercise certain movements (e.g., “mental exercising”) (#21). It has also been noted that VR can contribute to the development of the imagination, attention, or language learning through multi-sensory and cognitive stimulation. One of the most relevant cases mentioned by a participant is a boy eight years old with spastic tetraparesis. “He cannot walk, he has undeveloped verbal language, he has a developed receptive language but below his chronological age. He wishes to experience as many things and activities as possible, including those he is unable to do” (#5). The specialists consider that for these children this technology would help them to be closer to the places they want to see and the places that otherwise are inaccessible to them. In this way, the frustration determined by some disabilities would decrease significantly.

- Specialists’ proposals on how VR could be used in educating children with disabilities.

Nineteen of the thirty-one specialists interviewed in the focus groups were very optimistic about the potential that VR might have in the education of children that they are working with. They considered that all the pupils can benefit in one way or another from this technology. However, the didactic material must be adapted considering the limits determined by each child’s disability. One of the participants delineated how this technology could be used according to the type of disability as follows: for children with autistic spectrum disorders this technology could be used to create a routine, explaining that many children with this disability behave very well in familiar environments, but they experience difficulties in adapting to changes in environment; for children with mental disabilities, VR could improve the development of their imagination, by developing the capacity of creating new representations or ideas; for those children with locomotor disabilities, VR can help them to explore new places, with heavy access, as climbing mountains or walking stairs. He concluded that he could use VR for all his pupils: “this technology could be used very well by all the children that I work with, regardless of disability” (#26).

They made several proposals for applications that could make an important contribution to improving the educational process of children with disabilities:

(a) For example, in mathematics, VR can help to view some geometrical figures in space, draw some inner lines, and build new geometric figures.
(b) For other sciences, some applications that simulate the genesis of geological structures (e.g., mountains), the water cycle in nature, or help to explore the natural environment have been proposed. It was considered that an application created to learn how animals and plants live would be highly appreciated by pupils and very suitable for developing positive feelings and behaviors among them.
(c) Other examples concerned some applications that help users learn about the human body and understand how the internal organs work and the importance of hygiene, including the correct use of personal tools.
(d) A very interesting proposal aims to create an application through which children understand how the seasons and other natural phenomena work. Because some children do not remember enough information about the weather and natural phenomena from one season to another, a VR application that provides the possibility of multi-sensory stimulation could be very useful to help children understand the changes determined by the succession of seasons.
(e) Another idea is to create an application where pupils can exercise to overcome their fears and phobias, which are common symptoms in children with deficits, especially those with some forms of autism.

Regarding children with minor or moderate disabilities, participants agreed that “they need concrete and intuitive materials in the process of training and developing skills” (#29). It was proposed to make an application for presenting jobs or for exemplifying the operations required in a job. “It would be useful to have an application that would help them convey their professional expectations into real facts. It could be a great landmark in their professional orientation” (#25) said one of the participants. Another added these new arguments: “Definitely! Virtual reality brings into schools learning environments that are otherwise difficult to achieve. For example, VR can be used to train personal autonomy or practice a job” (#31).

### 4.3. (O3) Identifying Specialists’ Views on Children with Disabilities Who Are Best Suited to Use VR in Learning

The specialists agreed that VR could be used in the education process of children with disabilities in inclusive education. They identified categories of children with disabilities who could benefit from such technologies but also cases that are unsuitable for using VR were identified.

- Children with disabilities who can benefit from VR in the learning process

Among the pupils who might be receptive to using VR in the learning process might be included the following: children passionate about computers, phones, or other gadgets; children who are not anxious in the presence of new things; children capable of understanding information and receptive to using technology; and children whose cognitive areas are not greatly affected and they only have physical problems that affect their ability to move. In this regard, one of the participants stated: “Definitely yes, I can use VR for a certain category of children. For example, those who suffer from spastic tetraparesis, who have movement difficulties, but would like to visit some places, such as the mountains, even virtually” (#5).

- Children with disabilities for whom VR is not recommended

The participants made a series of remarks about situations when VR technology should not be used. They considered that each pupil should be analyzed independently, as VR may not be the appropriate education method for some of them. Children with epilepsy, claustrophobia, photosensitivity, or some children with a form of autism were mentioned. To support these opinions, some of the participants said the following: “Some of the children with a form of autism do not like to be touched, others have claustrophobia. For the children with epilepsy or photosensitivity it is dangerous” (#3); “It could be used in a better way in the case of children with mental moderate disabilities. Those with severe disabilities may experience problems with balance, dizziness, and coordination” (#24); “Unfortunately, many of the children we work with have severe, profound and associated impairments. So, this technology is not for them” (#2).

All the participants in the focus groups agreed that pupils with disabilities need concrete and intuitive tools to achieve and develop some skills needed to assure a sustainable inclusive education. Among these tools, VR represents a real opportunity for most of them.

## 5. Conclusions and Implications

Taking into account the results of the research, the authors consider that the objectives have been achieved and by disseminating the results a contribution is made to the body of knowledge in education.

The answer to the first question, regarding the notoriety of VR among specialists that work with pupils with disabilities, was that this new technology is not very familiar to educators and most of them had not used it until this interview was conducted. However, they were excited by the opportunity to use such technology in the teaching process. Thus, several benefits arising from the use of VR in inclusive education were highlighted: VR could improve the way children perceive information; VR could allow children to access some environments almost impossible for some of them to reach; VR could provide alternative ways of integrating information for children with disabilities. These conclusions are in line with other results presented in the literature [3,4,22,23,24]. Therefore, in response to the second research question, the experts agreed that the use of VR could be considered to benefit children with disabilities and suggested that it could be used as a complementary tool in the teaching process.

Thus, some applications were proposed for fields such as mathematics, geography, biology, hygiene, and adaptation to various life situations (transportation, accessibility, finding a job, etc.). Similar applications were also proposed in the specialty literature, which highlights the role of VR experiences in the improvement of the emotional and social adaptation skills of children with various disabilities [45,47,56,59].

Overall, discussions highlighted the connection between VR and educational progress in children with disabilities, but the respondents were cautious about replacing real experiences with VR ones, stressing that a real experience is preferable. The conclusion was that the VR-based applications should be tested before use. Thus, in the beginning, children would be selected by psychologists and those familiar with technology should be selected. Their exposure should be gradual, under the close supervision of a psychologist who is currently working with those children and who is able to assess their development. This conclusion represents the answer to the third research question, about the profile of children with disabilities for whom the use of VR in learning is suitable.

Taking into account all the ideas mentioned above, we consider that the answers to the research questions were obtained. Therefore, the use of VR as a teaching tool aimed at adapting the educational process to the needs of pupils could contribute to the progress of inclusive education. Even though VR is not yet commonly used by specialists who work with children with disabilities, they believe that it could help these children in the educational process. This conclusion is supported by the findings from the specialty literature which highlight that VR could help in inclusive education [35,36] as the learning environment would become more accessible for them [38,39]. Thus, VR could be considered a truly original tool for developing sustainable inclusive education. Compared to other technologies—such as watching images or movies—VR technology has an important advantage—it permits a very good immersion in the lesson context.

The results of this study have strong implications for several stakeholders: education institutions, the business environment, national education regulatory bodies, children with disabilities and their parents, etc. In our opinion, the design and implementation of VR applications in the process of educating children with disabilities must involve all these actors. At the academic level, through joint projects of the faculties with a profile in psychology and educational sciences, but also those with an IT profile, pilot programs must be put into practice to improve the teaching of children with different types of disabilities. To support these programs, at the business level, there is an opportunity to develop digital lessons that include VR technology. The companies will be in charge of developing the educational packages that are to be offered to the education institutions included in these pilot programs. The applications will be tested and validated by specialists in education but also by parents and the Ministry of Education. After validation, the programs could be extended to other schools and finally to the national level.

For its part, the Ministry of Education could further initiate a series of specific actions to promote the use of VR in the education of children with disabilities. Thus, all the actors could benefit: children and their parents could benefit from a better education; academia can conduct scientific research on the use of VR applications in the education of children with disabilities; companies could make a profit from selling applications; and finally, national bodies could contribute to the achievement of the goals related to the development of a sustainable inclusive education.

Additionally, integrating modern technologies in education has become more than necessary in the current context of the COVID-19 pandemic. Thus, Romania’s digital transformation becomes a priority, and the Ministry of Education has to transform the Romanian school into a modern and accessible one, based on new digital technologies. In this regard, the inclusion of VR technology in education, especially for children with disabilities, as a useful and modern tool in the educational process, could be a part of this digitization process.

Our study complements scientific knowledge validated by other research with new data on how VR can be used to educate children with disabilities and the potential opportunities and limitations of using this technology. From a scientific point of view, our study contributes to the development of education sciences by identifying a tool to help improve the teaching–learning process and make it more attractive and more socially sustainable. This tool could facilitate the teaching of curricular subjects and the development of social and cultural competences of this category of students, considered one of the most discriminated against in terms of access to education by UNICEF [21]. Thus, children with disabilities can benefit from digital learning materials that can help them to overcome the barriers they face, as highlighted in the specialty literature [18,19,20,21,22]. The research also has inherent limitations, primarily because only the opinions of specialists working with children with disabilities were identified. Future research should collect opinions from all stakeholders, such as parents of children with disabilities, secondary education specialists and teachers, academic researchers, IT specialists, central and local authorities, etc. Another future direction of research could focus on an analysis of the educational process regarding all potential users of VR, including students (with and without disabilities) and their parents. The results will be validated only after the VR applications are created and implemented in schools, and their results are measured independently. However, the research presented here is a necessary starting point for each new product to be launched into a market.

## Figures and Tables

**Table 1:** The characteristics of sample members.

The Respondent’s Position/Specialization	Code Number	Age	Years of Experience in Working with Special Needs Children
Speech therapist	1	62	32
Speech therapist	2	62	19
Speech therapist	3	56	20
Professor speech disorder therapy	4	47	11
Speech therapist	5	44	26
Speech therapist	6	31	5
Psychologist	7	47	28
Speech therapist	8	42	23
Special Education Teacher	9	45	12
Special Education Teacher	10	27	5
Special Education Teacher	11	46	20
Special Education Teacher	12	39	20
Special Education Teacher	13	45	10
Special Education Teacher	14	59	40
Special Education Teacher	15	31	9
Special Education Teacher	16	44	12
Professor of Physical Therapy	17	39	5
Itinerant/Support Teacher	18	54	17
Special Education Teacher/Teacher coordinator educators (coordinator of 47 itinerant/support teachers)	19	34	11
Social worker	20	39	10
Teacher of physical education and sport	21	40	15
Special Education Teacher	22	37	10
Professor of Physical Therapy	23	28	7
Special Education Teacher	24	45	13
Itinerant/Support teacher	25	40	11
Special Education Teacher/Itinerant/Support teacher	26	48	8
Special Education Teacher/Itinerant/Support teacher	27	38	11
Psychologist/Itinerant/Support teacher	28	32	9
Itinerant/Support teacher and parent of a child with Down syndrome	29	44	9
Itinerant/Support teacher	30	44	22
Special Education Teacher/Itinerant/Support teacher	31	34	9

## Data Availability

Data sharing is not applicable to this article.
